# Urban-rural disparities in the trends and causes of maternal deaths in Jinan, 1991–2025: a longitudinal trend analysis

**DOI:** 10.3389/fpubh.2026.1830524

**Published:** 2026-05-19

**Authors:** Xiaoxia Zheng, Jiaxue Pan, Qian Sun, Yangyang Tan, Xingrong Ma, Zhongliang Li

**Affiliations:** 1 Department of Gynecology, Jinan Maternity and Child Care Hospital Affiliated to Shandong First Medical University, Jinan, China; 2 Department of Women Healthcare, Jinan Maternity and Child Care Hospital Affiliated to Shandong First Medical University, Jinan, China; 3 Department of Health Education, Jinan Maternity and Child Care Hospital Affiliated to Shandong First Medical University, Jinan, China; 4 Department of Health Care, Jinan Maternity and Child Care Hospital Affiliated to Shandong First Medical University, Jinan, China

**Keywords:** causes, epidemiology, maternal deaths, trends, urban-rural disparities

## Abstract

**Background:**

Differences in health resources between rural and urban areas were identified as an important factor hindering the further reduction of maternal mortality ratio (MMR). However, few studies were conducted to analyze the trends and causes between them. Jinan in eastern China was selected as the research site, and differences and trends in maternal deaths from 1991 to 2025 were analyzed to provide suggestions for government departments to formulate targeted measures.

**Methods:**

Data such as the number of live births, maternal deaths, and causes of deaths were collected from paper files, electronic sheets, and web-based information platforms in Jinan. The study period was divided into seven stages with 5-year as a cycle, and Joinpoint Regression Model was used to analyze the trends of maternal deaths in urban and rural areas.

**Results:**

A total of 2,115,657 live births and 370 maternal deaths were included from 1991 to 2025 in Jinan. The overall MMR was 17.49 per 100,000 live births, of which the rural MMR was 21.15 per 100,000 live births and the urban MMR was 10.68 per 100,000 live births. The MMR showed an overall downward trend, with average annual percent change (AAPC) of −4.72% (95% CI: −5.32, −4.11), −4.03% (95% CI: −4.64, −3.41), and −5.49% (95% CI: −6.22, −4.75) in the total population, rural areas, and urban areas, respectively. The main causes of maternal deaths were obstetric hemorrhage (OH), hypertensive disorders of pregnancy (HDP), amniotic fluid embolism (AFE), heart disease (HD), venous thrombosis and pulmonary embolism (VTE), hepatopathy (Hep), and puerperal infection (PI). A total of 129 avoidable maternal deaths were recorded from 1991 to 2025, with a higher proportion in rural areas than in urban areas (85.27 vs. 14.73%). The main causes of avoidable deaths were Individual, Family and Community (46.51%) and Health Facility (43.41%).

**Conclusion:**

Some achievements had been made in Jinan in reducing MMR. Pregnant women in rural areas still faced challenges in controlling MMR. Efforts to reduce MMR, the government should take comprehensive measures to improve health awareness in rural areas and address the weak rescue capacity of rural hospitals.

## 1 Introduction

Maternal mortality ratio (MMR) was an important indicator for evaluating a country's socioeconomic development, medical service level, and women's health rights (1). It was also a crucial indicator that affects the population quality and reflects the social equity (2).

To reduce maternal deaths, the Millennium Development Goals (3) and the 2030 Sustainable Development Goals (4) were successively issued by the United Nations, and all countries were urged to take comprehensive measures to reduce maternal deaths. After years of efforts, the maternal mortality ratio has declined effectively across the world (5). However, many low- and middle-income countries have still failed to achieve the targets on schedule (6–9).

As a developing country with a large population, a series of measures were adopted to reduce maternal deaths in China. The MMR was effectively controlled (10–12), and China was ranked among countries with high performance in global maternal and child health (13).

Similar to the global challenges in reducing maternal deaths, the long-standing health disparities between rural and urban areas in China have also become an important factor hindering the reduction of maternal mortality (14–16).

Currently, there were few systematic analyses on the differences in maternal mortality between rural and urban areas. Moreover, research on policy recommendations based on these differences was insufficient, which made it difficult to provide a basis for further reducing MMR.

Jinan was a city in eastern China, whose rural-urban economic development, geographical characteristics and medical resource distribution were informative for studying rural-urban maternal health disparities.

Based on the surveillance data of maternal deaths in Jinan from 1991 to 2025, the trends and disparities in maternal deaths and theirs causes between urban and rural areas were systematically analyzed, and the major contributing factors were investigated. This study was expected to provide evidence for the government to optimize the allocation of health care resources and formulate targeted intervention strategies.

## 2 Methods

### 2.1 Study area and period

This was a retrospective survey conducted in Jinan, Shandong Province, from 1991 to 2025. As the capital of Shandong Province, Jinan has 15 districts and counties with a population of 9.4 million. Maternal mortality surveillance in Jinan has been implemented since 1991. After 2015, individual maternal death data were collected through a web-based information platform. Since 1996, “Twelve-Grid Table” released by WHO has been used to analyze avoidable factors related to maternal deaths. These efforts provided valid data for the present study.

### 2.2 Data sources

The number of live births and maternal deaths were derived from paper file registrations, electronic spreadsheets, and Jinan Maternal and Child Health Information Platform. The data collection and reporting method was consistent with mainstream domestic studies: hospitals where maternal deaths occurred reported to district/county maternal and child health institutions, which reviewed and submitted the data to municipal maternal and child health institutions (17).

The causes of maternal death and influencing factors of avoidable deaths were obtained from individual case review data of maternal death. Experts organized by municipal maternal and child health institutions analyzed the main influencing factors of avoidable deaths using the “Twelve-Grid Table” released by WHO. According to World Health Organization, maternal mortality was considered to be caused by the interaction of three levels (Individual, Family and Community, Health Facility, and Health System and Policy Level) and four factors (Knowledge and Skills, Attitude, Resources, and Management Systems). The Twelve-Grid Table was developed on this basis to identify factors contributing to maternal mortality. For instance, deaths caused by pregnant women's reluctance to seek hospital care were classified as individual attitude factors; deaths resulting from inappropriate clinical practice in medical institutions were attributed to health facility skill factors; and deaths caused by poor coordination between administrative departments were categorized as health system management factors (18).

In Jinan, a medical record review panel consisting of five to seven professors from multiple disciplines was established for maternal mortality review. Factors related to each maternal death were analyzed following the above procedure, and the most critical factor was identified as the avoidable factor for that death.

### 2.3 Definitions

Maternal deaths adopt the definition released by WHO, that is to say, the death of a woman from any cause related to or aggravated by pregnancy or its management (excluding accidental or incidental causes) during pregnancy and childbirth or within 42 days of termination of pregnancy, irrespective of the duration and site of the pregnancy (1).

Direct obstetric factors and indirect obstetric factors are classified according to the definition of the World Health Organization (WHO) (19). Direct obstetric deaths are those resulting from obstetric complications of the pregnant state (pregnancy, labor and puerperium), and from interventions, omissions, incorrect treatment, or from a chain of events resulting from any of the above. Indirect obstetric deaths are those maternal deaths resulting from previous existing disease or disease that developed during pregnancy and not due to direct obstetric causes but were aggravated by the physiologic effects of pregnancy.

Urban pregnant women were defined as those who had lived in urban areas for over 6 months, while rural pregnant women were identified as those who had resided in towns or villages for more than 6 months.

Maternal mortality ratio (MMR) was calculated as the number of maternal deaths per 100,000 live births.

The Rural-Urban Ratio (RUR) of maternal deaths was computed as:(rural maternal deaths, mortality rate, or composition ratio)/(urban maternal deaths, mortality rate, or composition ratio).

### 2.4 Quality control

Supported by the management of the three-tier maternal and child health network, the reporting of maternal mortality data was governed by a rigorous quality control procedure. Institutions where maternal deaths occurred were required to report cases to district and county maternal and child health institutions within 12 hours. After verification, district and county maternal and child health institutions were required to report to municipal maternal and child health institutions within 24 hours. Medical institutions were instructed monthly to screen for any maternal deaths in departments including obstetrics, gynecology, intensive care units and emergency departments through medical record review and the hospital information system (HIS). Quarterly, district-level maternal and child health institutions collaborated with district centers for disease control and prevention and district civil affairs bureaus to conduct under-reporting surveys by cross-checking maternal mortality records. Semiannually, municipal maternal and child health institutions conducted city-wide under-reporting investigations of maternal deaths through municipal centers for disease control and prevention, municipal medical record quality control centers and municipal civil affairs bureaus. Since 1991, the under-reporting rate of maternal deaths had been maintained below 0.35%.

### 2.5 Statistics

The overall study period was divided into seven consecutive non-overlapping phases with 5-year intervals.

Maternal Mortality Ratio, Proportion and Rural-Urban Ratio were applied to analyze rural-urban differences among different study stages.

Trend analysis was performed using the Joinpoint Regression Program (version 4.9.0.0). The Joinpoint regression model was fitted using weighted least squares to analyze the temporal trends of the above indicators in the surveillance area. The Permutation test was used to identify statistically significant joinpoints in the model. The annual percent change (APC) and the average annual percent change (AAPC), together with their 95% confidence intervals (95% CI), were calculated. If the 95% CI includes 0, it indicated that the AAPC trend change was not statistically significant. When conducting full-year analysis according to the time span, the maximum number of joinpoints in the model was set to 3. When analyzing different study periods, the number of joinpoints was set to 0.

## 3 Results

### 3.1 Trends of maternal deaths between urban and rural areas in Jinan

From 1991 to 2025, a total of 2,115,657 live births and 370 maternal deaths were included in this study. The overall MMR was 17.49 per 100,000 live births. Among them, the highest rate was recorded at 44.06 per 100,000 live births in 1991, and the lowest was 1.76 per 100,000 live births in 2025. A downward trend was observed, with the AAPC of −4.72% (95% CI: −5.32, −4.11).

Three inflection points were observed in the MMR trend from 1991 to 2025, namely 2000, 2010, and 2018. After each inflection point, the maternal mortality rate achieved a significant reduction: it was controlled within 30 per 100,000 live births after 2000, further controlled within 15 per 100,000 live births after 2010, and continuously controlled within 10 per 100,000 live births after 2018. The highest decline rate occurred during the period 1991–2000 (*APC*= −14.27%, 95% CI: −25.12, −3.13), followed by the period 2001–2010 (*APC* = −3.95%, 95% CI: −9.32, −1.17).

**Figure 1:**
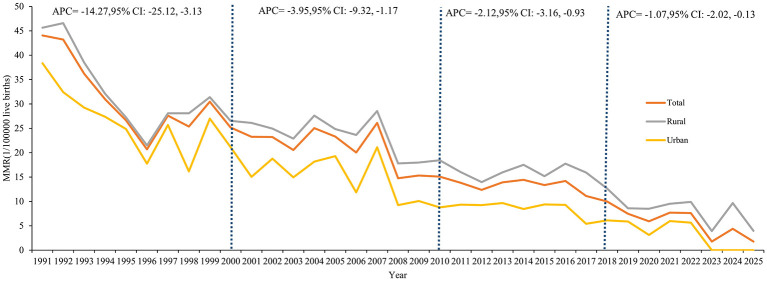
Trends of maternal deaths in urban and rural areas, 1991–2025.

Similarly, downward MMR trends were observed in both rural and urban areas. In rural areas, the MMR decreased from 45.66 per 100,000 live births in 1991 to 3.98 per 100,000 live births in 2025, with the AAPC of −4.03% (95% CI: −4.64, −3.41). In urban areas, it decreased from 38.35 per 100,000 live births in 1991 to 0 per 100,000 live births in 2023, with the AAPC of −5.49% (95% CI: −6.22, −4.75). Besides, maternal deaths occurred in rural areas every year over the 30-year period, while no maternal deaths were recorded in urban areas after 2022 (Table 1, Figure 1).

**Table 1:** Trends of maternal deaths in urban and rural areas, 1991–2025.

Year	Total			Rural			Urban		
	Births	Maternal deaths	MMR	Births	Maternal deaths	MMR	Births	Maternal deaths	MMR
1991	47,662	21	44.06	37,231	17	45.66	10,431	4	38.35
1992	39,329	17	43.23	30,075	14	46.55	9,254	3	32.42
1993	41,421	15	36.21	31,161	12	38.51	10,260	3	29.24
1994	45,237	14	30.95	34,267	11	32.10	10,970	3	27.35
1995	48,811	13	26.63	36,733	10	27.22	12,078	3	24.84
1996	53,149	11	20.70	41,888	9	21.49	11,261	2	17.76
1997	57,947	16	27.61	46,273	13	28.09	11,674	3	25.70
1998	55,154	14	25.38	42,769	12	28.06	12,385	2	16.15
1999	52,504	16	30.47	41,391	13	31.41	11,113	3	27.00
2000	55,618	14	25.17	41,451	11	26.54	14,167	3	21.18
2001	51,590	12	23.26	38,281	10	26.12	13,309	2	15.03
2002	56,036	13	23.20	40,070	10	24.96	15,966	3	18.79
2003	68,110	14	20.55	48,055	11	22.89	20,055	3	14.96
2004	59,962	15	25.02	43,439	12	27.62	16,523	3	18.16
2005	55,791	13	23.30	40,240	10	24.85	15,551	3	19.29
2006	54,888	11	20.04	38,059	9	23.65	16,829	2	11.88
2007	57,501	15	26.09	38,538	11	28.54	18,963	4	21.09
2008	60,974	9	14.76	39,330	7	17.80	21,644	2	9.24
2009	58,690	9	15.33	38,884	7	18.00	19,806	2	10.10
2010	66,206	10	15.10	43,377	8	18.44	22,829	2	8.76
2011	65,022	9	13.84	43,652	7	16.04	21,370	2	9.36
2012	64,621	8	12.38	43,001	6	13.95	21,620	2	9.25
2013	64,621	9	13.93	43,894	7	15.95	20,727	2	9.65
2014	69,344	10	14.42	45,703	8	17.50	23,641	2	8.46
2015	67,472	9	13.34	46,134	7	15.17	21,338	2	9.37
2016	77,395	11	14.21	45,053	8	17.76	32,342	3	9.28
2017	81,120	9	11.09	44,006	7	15.91	37,114	2	5.39
2018	80,130	8	9.98	47,370	6	12.67	32,760	2	6.11
2019	80,528	6	7.45	46,531	4	8.60	33,997	2	5.88
2020	67,329	4	5.94	35,271	3	8.51	32,058	1	3.12
2021	65,004	5	7.69	31,500	3	9.52	33,504	2	5.97
2022	65,494	5	7.63	30,251	3	9.92	35,243	2	5.67
2023	56,107	1	1.78	25,632	1	3.90	30,475	0	0.00
2024	68,157	3	4.40	31,002	3	9.68	37,155	0	0.00
2025	56,733	1	1.76	25,123	1	3.98	31,610	0	0.00
Total	21,15,657	370	17.49	13,75,635	291	21.15	7,40,022	79	10.68
AAPC (95% CI)	−4.72 (−5.32, −4.11)			−4.03 (−4.64, −3.41)			−5.49 (−6.22, −4.75)		

**Figure 2:**
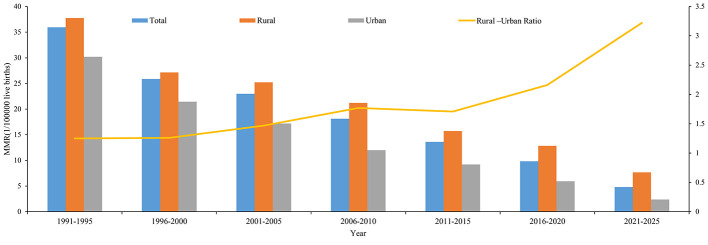
Maternal deaths differences of urban and rural areas in Jinan among different study phases.

The highest MMR in the all pregnant women was 35.96 per 100,000 live births in 1991–1995, and the lowest was 4.82 per 100,000 live births in 2021–2025. The trends of MMR in rural and urban areas showed a downward trend across different study phases. The MMR in rural areas was consistently higher than that in urban areas across all study phases, with an overall MMR rural-urban ratio of 1.98. Among the different study phases, the MMR Rural-Urban Ratio was gradually increased from 1.25 in 1991–1995 to 3.22 in 2021–2025 (Table 2, Figure 2).

**Table 2:** Maternal deaths differences of urban and rural areas in jinan among different study phases.

Year	Total			Rural			Urban			Rural–urban ratio (95% CI)
	Births	Maternal deaths	MMR	Births	Maternal deaths	MMR	Births	Maternal deaths	MMR	
1991–1995	22,2460	80	35.96	1,69,467	64	37.77	5,2993	16	30.19	1.25 (0.44, 2.94)
1996–2000	27,4372	71	25.88	2,13,772	58	27.13	60,600	13	21.45	1.26 (0.43, 2.95)
2001–2005	29,1489	67	22.99	2,10,085	53	25.23	81,404	14	17.20	1.47 (0.22, 3.16)
2006–2010	29,8259	54	18.11	1,98,188	42	21.19	1,00,071	12	11.99	1.77 (0.08, 3.46)
2011–2015	33,1080	45	13.59	2,22,384	35	15.74	1,08,696	10	9.20	1.71 (0.02, 3.40)
2016–2020	38,6502	38	9.83	2,18,231	28	12.83	1,68,271	10	5.94	2.16 (0.47, 3.85)
2021–2025	31,1495	15	4.82	1,43,508	11	7.67	1,67,987	4	2.38	3.22 (1.53, 4.91)
Total	21,15,657	370	17.49	13,75,635	291	21.15	7,40,022	79	10.68	1.98 (1.20, 2.47)
AAPC (95% CI)	−26.16 (−32.34, −19.41)			−21.43 (−25.97, −16.61)			−32.04 (−39.01, −24.27)			15.64 (8.86, 22.83)

### 3.2 Differences of maternal deaths causes in in all pregnant women among different study phases in Jinan

The rank of maternal death causes from 1991 to 2025 was obstetric hemorrhage (OH), hypertensive disorders of pregnancy (HDP), amniotic fluid embolism (AFE), heart disease (HD), venous thrombosis and pulmonary embolism (VTE), hepatopathy (Hep) and puerperal infection (PI). Among them, the incidence of OH was 4.49 per 100,000 live births with a proportion of 25.68%, and the incidence of HDP was 2.84 per 100,000 live births with a proportion of 16.22%.

Viewed from changes in the rank of maternal death causes across different study phases, a significant downward trend was observed in AFE, which has been lower than HD and VTE since 2006. HDP also showed a gradual downward trend and has been lower than heart disease since 2016. Although maternal deaths due to OH have significantly decreased, its ranking has remained the leading cause.

With respect to changes in the incidence of maternal death causes among different phases, the specific mortality rates of obstetric factors such as OH, HDP, AFE, and PI have all decreased to varying degrees. Among them, OH had the largest AAPC [−32.77%, 95% CI: (−36.99, −28.27)], and PI had the most obvious downward effect, with no cases recorded since 2006. Among indirect obstetric factors, aside from a decrease in the mortality rate of Hep, no other diseases showed a significant trend.

**Figure 3:**
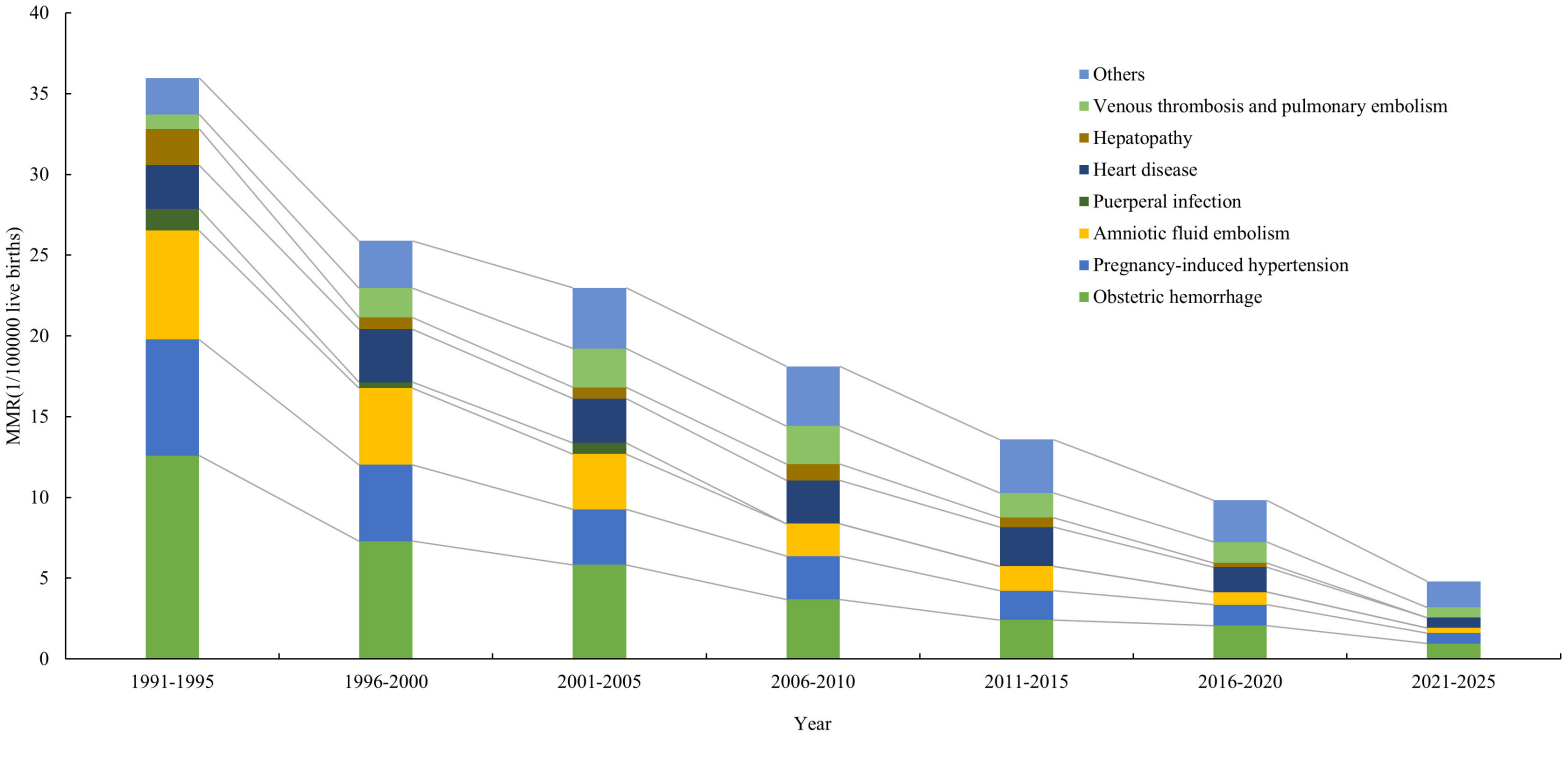
Differences of maternal deaths causes in Jinan among different study phases.

Given changes in the proportion of maternal death causes in different phases, the proportions of OH and HDP were relatively high and showed no obvious changing trend. AFE showed a downward trend with the AAPC of −16.59% (95% CI: −19.94, −13.10); the proportions of indirect obstetric factors mostly showed an upward trend, among which the proportions of VTE, HD and other factors increased with an AAPC of 25.38% (95% CI: 3.66, 51.65), 9.58% (95% CI: 1.25, 21.60) and 28.93% (95% CI: 18.02, 40.86), respectively (Table 3, Figure 3).

**Table 3:** Differences of maternal deaths causes in Jinan among different study phases.

Year	Obstetric hemorrhage		Hypertensive disorders of pregnancy		Amniotic fluid embolism		Puerperal infection		Heart disease		Hepatopathy		Venous thrombosis and pulmonary embolism		Others	
	MMR	Propor-tion	MMR	Propor-tion	MMR	Propor-tion	MMR	Propor-tion	MMR	Propor-tion	MMR	Propor-tion	MMR	Propor-tion	MMR	Propor-tion
1991–1995	12.59	35.00	7.19	20.00	6.74	18.75	1.35	3.75	2.70	7.50	2.25	6.25	0.90	2.50	2.25	6.25
1996–2000	7.29	28.17	4.74	18.31	4.74	18.31	0.36	1.41	3.28	12.68	0.73	2.82	1.82	7.04	2.92	11.27
2001–2005	5.83	25.37	3.43	14.93	3.43	14.93	0.69	2.99	2.74	11.94	0.69	2.99	2.40	10.45	3.77	16.42
2006–2010	3.69	20.37	2.68	14.81	2.01	11.11	0.00	0.00	2.68	14.81	1.01	5.56	2.35	12.96	3.69	20.37
2011–2015	2.42	17.78	1.81	13.33	1.51	11.11	0.00	0.00	2.42	17.78	0.60	4.44	1.51	11.11	3.32	24.44
2016–2020	2.07	21.05	1.29	13.16	0.78	7.89	0.00	0.00	1.55	15.79	0.26	2.63	1.29	13.16	2.59	26.32
2021–2025	0.96	20.00	0.64	13.33	0.32	6.67	0.00	0.00	0.64	13.33	0.00	0.00	0.64	13.33	1.61	33.33
Total	4.49	25.68	2.84	16.22	2.51	14.32	0.28	1.62	2.22	12.70	0.71	4.05	1.56	8.92	2.88	16.49
AAPC (95% CI)	−32.77 (−36.99, −28.27)	−8.92 (−14.64, 2.83)	−6.86 (−9.98, −3.64)	−7.82 (−8.38, 2.64)	−38.41 (−44.51, −31.65)	−16.59 (−19.94, −13.10)	/	/	−19.12 (−32.14, 3.60)	9.58 (1.25, 21.60)	−26.96 (−45.18, −2.69)	−6.48 (−27.94, 21.37)	−7.47 (−27.62, 18.29)	25.38 (3.66, 51.65)	−4.78 (−18.19, 10.82)	28.93 (18.02, 40.86)

### 3.3 Differences of maternal deaths causes in rural pregnant women among different study phases in Jinan

The rank of maternal death causes in rural areas was OH, HDP, AFE, HD, VTE, Hep, and PI. Among them, the MMR due to OH was 5.89 per 100,000 live births with a proportion of 27.84%, and the MMR due to HDP was 3.85 per 100,000 live births with a proportion of 18.21%.

The changes in the ranking of maternal death causes across different study phases in rural areas were consistent with those in all pregnant women. Specifically, among obstetric factors, HDP and AFE showed a gradual downward trend, while among indirect obstetric factors, HD and VTE showed a gradual upward trend.

The trend of MMR in rural areas across different phases was consistent with that in the total pregnant women, that was, the MMR of obstetric factors gradually decreased, while that of indirect obstetric factors did not decrease. In terms of the decline rate of MMR from various diseases, AFE showed the highest AAPC at −30.26% (95% CI: −40.20, −18.66), followed by HDP at −28.61% (95% CI: −38.18, −17.55), and OH at −24.60% (95% CI: −27.75, −21.32). The AAPC for these three diseases in rural areas were all lower than the corresponding urban rates (−34.38% (95% CI: −50.39, −13.20), −29.38% (95% CI: −47.28, −0.02), and −45.55% [95% CI: −58.87, −27.90)], respectively.

Regarding the proportional distribution of maternal deaths in rural areas, the pattern was similar to that in all pregnant women: the proportion of HDP and AFE decreased, while those of HD and VTE increased. The AAPC in HDP [−1.91%, 95% CI (−3.79, −0.01)] was lower in rural areas than in urban areas. In contrast, the AAPC in VTE was 25.38% [25.25% (4.88, 36.76)] in rural areas, higher than that in urban areas (17.76%; Table 4).

**Table 4:** Differences of maternal deaths causes in rural areas among different study phases.

Year	Obstetric hemorrhage		Hypertensive disorders of pregnancy		Amniotic fluid embolism		Puerperal infection		Heart disease		Hepatopathy		Venous thrombosis and pulmonary embolism		Others	
	MMR	Propor-tion	MMR	Propor-tion	MMR	Propor-tion	MMR	Propor-tion	MMR	Propor-tion	MMR	Propor-tion	MMR	Propor-tion	MMR	Propor-tion
1991–1995	12.98	34.38	7.67	20.31	7.08	18.75	1.77	4.69	2.95	7.81	2.95	7.81	0.59	1.56	1.77	4.69
1996–2000	7.95	29.31	5.61	20.69	4.21	15.52	0.47	1.72	2.81	10.34	0.94	3.45	1.87	6.90	3.27	12.07
2001–2005	6.66	26.42	4.28	16.98	4.28	16.98	0.95	3.77	3.33	13.21	0.48	1.89	2.38	9.43	2.86	11.32
2006–2010	5.05	23.81	3.53	16.67	2.52	11.90	0.00	0.00	3.03	14.29	1.51	7.14	3.03	14.29	2.52	11.90
2011–2015	3.60	22.86	2.70	17.14	2.25	14.29	0.00	0.00	2.25	14.29	0.90	5.71	2.25	14.29	1.80	11.43
2016–2020	3.21	25.00	2.29	17.86	0.92	7.14	0.00	0.00	1.37	10.71	0.00	0.00	1.83	14.29	3.21	25.00
2021–2025	2.09	27.27	0.70	9.09	0.00	0.00	0.00	0.00	1.39	18.18	0.00	0.00	0.70	9.09	2.79	36.36
Total	5.89	27.84	3.85	18.21	3.05	14.43	0.44	2.06	2.47	11.68	0.95	4.47	1.89	8.93	2.62	12.37
AAPC (95% CI)	−24.60 (−27.75, −21.32)	−0.82 (−1.93, 0.3)	−28.61 (−38.18, −17.55)	−1.91 (−3.79, −0.01)	−30.26 (−40.20, −18.66)	−3.05 (−5.77, −0.26)	/	/	−13.58 (−22.32, −3.86)	1.94 (0.03, 3.94)	−17.31 (−59.04, 66.96)	−0.21 (−12.64, 14.93)	1.49 (−27.48, 42.03)	25.25 (4.88, 36.76)	3.14 (−9.53, 17.58)	5.59 (2.40, 8.87)

### 3.4 Differences of maternal deaths causes in urban pregnant women among different study phases in Jinan

The rank of maternal death causes in urban areas was OH, HD, AFE, VTE, HDP and Hep. Among them, the rank of HD and VTE were both higher than those in rural areas.

Regarding changes in the rank order of maternal death causes across various study phases, urban areas differed from rural areas. OH and HDP showed a downward trend, and both fell below HD after 2006. Since then, HD has become the leading cause of maternal death in urban areas.

In terms of cause-specific mortality across phases, the reduction in direct obstetric causes was substantially greater in urban than in rural areas. Specifically, only one case each of OH and HDP, and only two cases of AFE, have been recorded since 2011.

With respect to the proportion of maternal deaths causes, urban areas again differed from rural areas. OH decreased markedly in urban areas, with an annual average decline of −24.76% (95% CI: −43.60, −0.38). By contrast, AFE showed no obvious downward trend and remained sporadic. Among indirect obstetric causes, HD increased sharply, with the AAPC of 31.10% (95% CI: 8.53, 87.90), considerably higher than the 9.58% observed in rural areas (Table 5).

**Table 5:** Differences of maternal deaths causes in urban areas among different study phases.

Year	Obstetric hemorrhage		Hypertensive disorders of pregnancy		Amniotic fluid embolism		Puerperal infection		Heart disease		Hepatopathy		Venous thrombosis and pulmonary embolism		Others	
	MMR	Propor-tion	MMR	Propor-tion	MMR	Propor-tion	MMR	Propor-tion	MMR	Propor-tion	MMR	Propor-tion	MMR	Propor-tion	MMR	Propor-tion
1991–1995	11.32	37.50	5.66	18.75	5.66	18.75	0.00	0.00	1.89	6.25	0.00	0.00	1.89	6.25	3.77	12.50
1996–2000	4.95	23.08	1.65	7.69	6.60	30.77	0.00	0.00	4.95	23.08	0.00	0.00	1.65	7.69	1.65	7.69
2001–2005	3.69	21.43	1.23	7.14	1.23	7.14	0.00	0.00	1.23	7.14	1.23	7.14	2.46	14.29	6.14	35.71
2006–2010	1.00	8.33	1.00	8.33	1.00	8.33	0.00	0.00	2.00	16.67	0.00	0.00	1.00	8.33	6.00	50.00
2011–2015	0.00	0.00	0.00	0.00	0.00	0.00	0.00	0.00	2.76	30.00	0.00	0.00	0.00	0.00	6.44	70.00
2016–2020	0.59	10.00	0.00	0.00	0.59	10.00	0.00	0.00	1.78	30.00	0.59	10.00	0.59	10.00	1.78	30.00
2021–2025	0.00	0.00	0.60	25.00	0.60	25.00	0.00	0.00	0.00	0.00	0.00	0.00	0.60	25.00	0.60	25.00
Total	1.89	17.72	0.95	8.86	1.49	13.92	0.00	0.00	1.76	16.46	0.27	2.53	0.95	8.86	3.38	31.65
AAPC (95% CI)	−45.55 (−58.87, −27.90)	−24.76 (−43.60, −0.38)	−29.38 (−47.28, −0.02)	−10.85 (−27.19, −68.76)	−34.38 (−50.39, −13.20)	−2.54 (−32.36, 40.43)	/	/	−4.38 (−32.37, 35.20)	31.10 (8.53, 87.90)	/	/	−20.73 (−32.97, −6.25)	17.76 (−3.50, 43.70)	−17.29 (−45.92, 26.51)	21.6 (−13.63, 71.19)

### 3.5 Differences of the maternal death rural-urban ratio among different study phases in Jinan

Most rural-urban ratio of MMR and for the proportions of maternal death causes exceeded 1. Only the rural-urban ratio for the proportion of HD was below 1. In addition, the rural-urban ratios of both MMR and proportion of maternal deaths due to other causes were less than 1 (Table 6).

**Table 6:** Differences of rural –urban ratio between rural areas and urban areas among different study phases.

Year	Obstetric hemorrhage		Hypertensive disorders of pregnancy		Amniotic fluid embolism		Heart disease		Hepatopathy		Venous thrombosis and pulmonary embolism		Others	
	MMR	Propor-tion	MMR	Propor-tion	MMR	Propor-tion	MMR	Propor-tion	MMR	Propor-tion	MMR	Propor-tion	MMR	Propor-tion
1991–1995	1.15	0.92	1.36	1.08	1.25	1.00	1.56	1.25	/	/	0.31	0.25	0.47	0.38
1996–2000	1.61	1.27	3.40	2.69	0.64	0.50	0.57	0.45	/	/	1.13	0.90	1.98	1.57
2001–2005	1.81	1.23	3.49	2.38	3.49	2.38	2.71	1.85	0.39	0.26	0.97	0.66	0.46	0.32
2006–2010	5.05	2.86	3.53	2.00	2.52	1.43	1.51	0.86	/	/	3.03	1.71	0.42	0.24
2011–2015	/	/	/	/	/	/	0.81	0.48	/	/	/	/	0.28	0.16
2016–2020	5.40	2.50	/	/	1.54	0.71	0.77	0.36	0.00	0.00	3.08	1.43	1.80	0.83
2021–2025	/	/	1.17	0.36	0.00	0.00	/	/	/	/	1.17	0.36	4.68	1.45
Total	3.11	1.57	4.07	2.06	2.05	1.04	1.41	0.71	3.50	1.76	2.00	1.01	0.77	0.39

### 3.6 Differences of avoidable factors of maternal deaths among different study phases in Jinan

From 1996 to 2025, a total of 129 maternal deaths were identified as avoidable, accounting for 44.48% of all maternal deaths. Of these, 110 cases occurred among rural women, representing 85.27% of all avoidable deaths.

**Figure 4:**
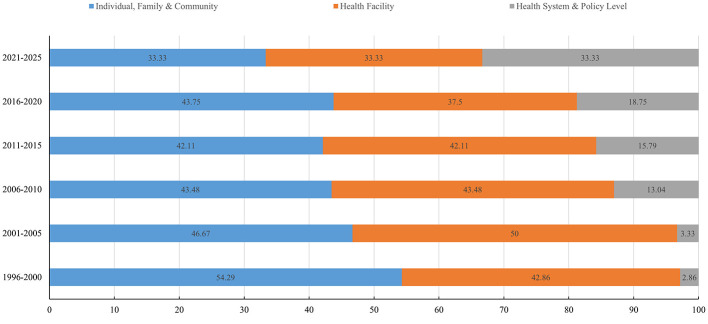
Differences of avoidable factors of maternal deaths among different study phases.

Individual, Family and Community factors were the leading avoidable causes (46.51%), followed by Health Facility factors (43.41%). The trends across the study phases showed a progressive increase in the proportion of social management factors, becoming the primary avoidable cause during 2021–2025 (Table 7, Figure 4).

**Table 7:** Differences of avoidable factors of maternal deaths among different study phases.

Factors	1996–2000		2001–2005		2006–2010		2011–2015		2016–2020		2021–2025		Total	
	Number	Propor-tion	Number	Propor-tion	Number	Propor-tion	Number	Propor-tion	Number	Propor-tion	Number	Propor-tion	Number	Propor-tion
Individual, family and community	19	54.29	14	46.67	10	43.48	8	42.11	7	43.75	2	33.33	60	46.51
Knowledge and skills	9	25.71	7	23.33	6	26.09	5	26.32	5	31.25	2	33.33	34	26.36
Attitude	5	14.29	3	10.00	2	8.70	1	5.26	0	0	0	0.00	11	8.53
Resources	4	11.43	4	13.33	2	8.70	2	10.53	2	12.5	0	0.00	14	10.85
Management systems	1	2.86	0	0.00	0	0.00	0	0.00	0	0	0	0.00	1	0.78
Health facility	15	42.86	15	50.00	10	43.48	8	42.11	6	37.5	2	33.33	56	43.41
Knowledge and skills	8	22.86	9	30.00	7	30.43	5	26.32	5	31.25	2	33.33	36	27.91
Attitude	2	5.71	1	3.33	0	0.00	0	0.00	0	0	0	0.00	3	2.33
Resources	3	8.57	3	10.00	2	8.70	0	0.00	0	0	0	0.00	8	6.20
Management systems	2	5.71	2	6.67	1	4.35	3	15.79	1	6.25	0	0.00	9	6.98
Health system and policy level	1	2.86	1	3.33	3	13.04	3	15.79	3	18.75	2	33.33	13	10.08
Knowledge and skills^*^	0	0.00	0	0.00	0	0.00	0	0.00	0	0	0	0.00	0	0.00
Attitude	0	0.00	0	0.00	0	0.00	0	0.00	0	0	0	0.00	0	0.00
Resources	0	0.00	0	0.00	0	0.00	0	0.00	0	0	0	0.00	0	0.00
Management systems	1	2.86	1	3.33	3	13.04	3	15.79	3	18.75	2	33.33	13	10.08

^*^AAPC=65.60, 95% CI (32.87, 106.39).

## 4 Discussion

This study found that maternal mortality in Jinan showed an overall downward trend from 1991 to 2025, which was consistent with the global trend. The average annual decline over the 30-year period was 4.72%, which was better than the global average and met the targets of the UN 2030 Sustainable Development Goals (4).

In 2021–2025, the MMR dropped to 4.82 per 100 000 live births. In urban areas, the MMR fell to 2.38 per 100 000 live births. These data have reached or been closed to the levels of Germany (4 per 100,000 live births), Poland (2 per 100,000 live births), the European Union (5 per 100,000 live births), Spain (3 per 100,000 live births), and Japan (3 per 100,000 live births) (2).

The downward trend was divided into four stages by three inflection points: 2001, 2010, and 2018. As these 3 years coincided with the introduction of three key interventions (Project for Reducing Maternal Mortality and Eliminating Neonatal Tetanus, Basic Public Health Services Program and Five Systems for Maternal and Infant Safety) in Jinan, it was inferred that the observed changes in maternal mortality were attributable to the implementation of these measures. Firstly, the Project for Reducing Maternal Mortality and Eliminating Neonatal Tetanus was launched in 2000. A key component of this program was to provide subsidies for hospital delivery for pregnant women, which increased the hospital delivery rate in China. Previous studies have shown that the hospital delivery rate reached 95.4% after the implementation of the program (20). The promotion of hospital delivery enables timely management of obstetric complications such as obstetric hemorrhage, thereby effectively reducing the maternal mortality ratio (21). Our data also showed obvious declines in obstetric hemorrhage after 2001. Secondly, the National Basic Public Health Services Program was introduced in 2009 (22). It provided five free prenatal medical examination and free postpartum medical care for all pregnant women. These services improved the management of HDP, gestational anemia, puerperal infection, and other complications (23). In our cause-specific analysis, no deaths from puerperal infection were recorded after 2006–2010. Thirdly, Five Systems for Maternal and Infant Safety was implemented in 2017 (24). Based on the policy, risk screening for pregnant women and referral treatment between hospitals were put forward, so high-risk pregnant women can be identified and intervened early, and timely treatment also can be provided to pregnant women with severe complications. In summary, increased national financial investment and improved clinical capacity in medical institutions were major factors responsible for the sustained decline in maternal mortality in Jinan.

In the analysis of the differences of MMR between rural and urban areas, we found that although MMR in both areas showed a downward trend, the rural-urban ratio of it gradually widened. In addition, most avoidable maternal deaths occurred in rural areas. Overall, the situation of preventing and controlling maternal deaths in rural areas was still severe. Similar conclusions had been drawn in other domestic and foreign studies (25), and the main reasons were as follows. Firstly, pregnant women in rural areas had lower health literacy than urban ones. A Chinese survey conducted in 2020 showed that the health literacy rate of rural pregnant women was only 47.81% of that in urban areas (26). In our analysis of avoidable factors in maternal deaths, we also found that during the period 1996–2000, when maternal deaths were relatively high, Individual, Family and Community accounted for 54.29% of all avoidable factors. In addition, it was also found that the rural-urban ratio of maternal deaths caused by HDP was the highest. Relevant studies have shown that the prevention and treatment of HDP were closely associated with weight management and blood pressure management during pregnancy, and the effective implementation of these tasks was closely related to the self-care awareness of pregnant women (27–29). The higher rural-urban ratio also reflected the weak health awareness among rural pregnant women. Secondly, it should be attributed to the insufficient health service capacity of medical institutions. The long-standing rural-urban economic system in China had led to the medical treatment capacity of township hospitals being significantly lower than that in urban areas (30). Most studies also point out that strengthening the development of primary medical institutions was the key measure to reduce maternal mortality (31, 32). In our study, avoidable maternal deaths caused by insufficient hospital service capacity were accounted for 27.91%, the highest proportion among all avoidable factors. At present, with the continuous deepening of China's urbanization process, more and more pregnant women have moved to cities, and the impact of the rural-urbandual economic system has been decreasing (33). However, relevant studies have shown that the remaining pregnant women in rural areas currently have weaker health awareness and lower economic income than before, and they face a higher risk of maternal death (34, 35). Therefore, more attention needs to be paid to the health of rural pregnant women in the future.

We found that obstetric hemorrhage was the leading cause of maternal death. Further analysis revealed that most such deaths occurred in rural areas, and it had remained the primary cause of maternal mortality in rural regions for 30 years. Numerous studies have demonstrated that the vast majority of deaths from obstetric hemorrhage are avoidable (36–38). Therefore, future efforts to reduce maternal mortality should focus on rural areas and further improve the capacity of primary-level institutions in estimating blood loss and emergency care for pregnant women.

In the maternal deaths rank analysis, indirect obstetric factors such as VTE and HD were gradually increasing. It was generally consistent with that observed in developed countries (39) (40, 41). The main reasons were as follows: firstly, it was related to the increase in older pregnant women. Following the implementation of the selective two-child policy in 2013 and the universal two-child policy in 2016 in China, the number of older pregnant women increased continuously and slowed down only after 2020 (42, 43). Advanced maternal age has been confirmed in many studies as an independent risk factor for pregnancy-related comorbidities and complications (44). The increasing proportions of HD and VTE among maternal death causes were observed during 2011–2020, which coincided with the rise in the older pregnant women after the relaxation of fertility policies. Secondly, it was associated with the insufficient multidisciplinary treatment capacity in medical institutions. Early pregnancy symptoms of heart disease and VTE were mostly non-specific, requiring high ability of early identification by obstetricians (45, 46). The management of these conditions requires not only multidisciplinary cooperation within hospitals but also participation from other government departments for hospitals with limited specialties. In our study, it was also found that maternal deaths caused by other indirect obstetric factors had been increasing year by year and had become the leading cause of maternal deaths. Similar to other studies, this also indicated that the etiology of maternal death had gradually expanded from a single disease to more complex and diverse pregnancy complicated with medical diseases (47). The prevention of such diseases required not only attention within the medical system but also support from other non-medical sectors such as charitable assistance and social care. In our analysis of the causes of avoidable maternal deaths, it was also found that the proportion of social sector management factors was increasing. Therefore, the focus of maternal death prevention and control in future should not only focus on efforts within the medical system but also arouse the whole society's attention to pregnant women.

This study had some advantages. Trends in maternal mortality rate, cause-of-death ranking, and causes of death among urban and rural pregnant women were longitudinally analyzed, and the reasons for these changes were also explored. The findings may provide useful references for reducing maternal mortality in other regions. In addition, the effectiveness of health policies implemented by government departments over the years was evaluated, and our analyses may offer valuable insights for government departments to formulate targeted policies.

The limitations of this study were that quality control could hardly be conducted on data with a long research period, which affected the accurate calculation of maternal mortality rate to a certain extent. In addition, the analysis of some maternal death causes was not based on high-quality evidence from prospective cohort studies, which was expected to be improved in further research.

## 5 Conclusion

Some achievements had been made in Jinan in the process of reducing maternal mortality. To further reducing the MMR, more attention should be paid to improving the capacity of primary health care institutions in rural areas and enhancing the health awareness of impoverished pregnant women in rural regions. Comprehensive measures should be taken by health administrative departments in collaboration with finance, charity and other relevant administrative departments to ensure maternal and infant safety.

## Data Availability

The original contributions presented in the study are included in the article/supplementary material, further inquiries can be directed to the corresponding author.

## References

[1] World Health Organization. Maternal Mortality Ratio. Geneva: World Health Organization (2023). Available online at: https://www.who.int/data/gho/data/themes/topics/indicator-groups/indicator-group-details/GHO/m-maternal-mortality (Accessed January 08, 2025).

[2] United Nations Population Fund. Trends in Maternal Mortality 2000–2023. New York, NY: UNPF. Available online at: https://www.unfpa.org/publications/trends-maternal-mortality-2000-2023 (Accessed April 15, 2025).

[3] United Nations. Millennium Declaration. New York, NY: United Nations.(2000). Available online at: https://undocs.org/en/A/RES/55/2 (Accessed September 18, 2000).

[4] United Nations. Transforming our world: the 2030 agenda for sustainable development. New York, NY: United Nations (2015).

[5] Ahmed S Ali M Shah I Tsui A. Effect of maternity care improvement, fertility decline, and contraceptive use on global maternal mortality reduction between 2000 and 2023: results from a decomposition analysis. Lancet Global health. (2026) 14:e33–48. doi: 10.1016/S2214-109X(25)00409-7 41192459 PMC12689492

[6] Sharma B Smith R Sharma BB Pennell C. Maternal mortality ratios in low- and middle-income countries: a comparison of estimation methods and relationships with sociodemographic covariates. AJOG Global Reports. (2025) 5:100438. doi: 10.1016/j.xagr.2024.100438 40224305 PMC11994042

[7] Collaborators GHAaQ. Measuring performance on the Healthcare Access and Quality Index for 195 countries and territories and selected subnational locations: a systematic analysis from the global burden of disease study 2016. Lancet (London, England) (2018) 391:2236–71. 29893224 10.1016/S0140-6736(18)30994-2 PMC5986687

[8] Boerma T Campbell OMR Amouzou A Blumenberg C Blencowe H Moran A . Maternal mortality, stillbirths, and neonatal mortality: a transition model based on analyses of 151 countries. Lancet Global Health. (2023) 11:e1024–31. doi: 10.1016/S2214-109X(23)00195-X 37349032 PMC10299966

[9] Kurjak A Stanojević M Dudenhausen J. Why maternal mortality in the world remains tragedy in low-income countries and shame for high-income ones: will sustainable development goals (SDG) help? J Perinat Med. (2023) 51:170–81. doi: 10.1515/jpm-2022-0061 35636412

[10] Collaborators GMM. Global, regional, and national levels of maternal mortality, 1990-2015: a systematic analysis for the global burden of disease Study 2015. Lancet (London, England). (2016) 388:1775–812. 27733286 10.1016/S0140-6736(16)31470-2 PMC5224694

[11] Liang J Li X Kang C Wang Y Kulikoff XR Coates MM . Maternal mortality ratios in 2852 Chinese counties, 1996-2015, and achievement of millennium development goal 5 in China: a subnational analysis of the global burden of disease study 2016. Lancet (London, England). (2019) 393:241–52. doi: 10.1016/S0140-6736(18)31712-4 30554785 PMC6336935

[12] Qiao J Wang Y Li X Jiang F Zhang Y Ma J . A lancet commission on 70 years of women's reproductive, maternal, newborn, child, and adolescent health in China. Lancet (London, England). (2021) 397:2497–536. doi: 10.1016/S0140-6736(20)32708-2 34043953

[13] World Health Organization. Global Status Report on Maternal Health 2023. Geneva: World Health Organization. (2023).

[14] Ward ZJ Atun R King G Dmello BS Goldie SJ. Global maternal mortality projections by urban/rural location and education level: a simulation-based analysis. EClinicalMedicine. (2024) 72:102653. doi: 10.1016/j.eclinm.2024.102653 38800798 PMC11126824

[15] Lawrence ER Klein TJ Beyuo TK. Maternal mortality in low and middle-income countries. Obstet Gynecol Clin North Am. (2022) 49:713–33. doi: 10.1016/j.ogc.2022.07.001 36328676

[16] Joseph KS Boutin A Lisonkova S Muraca GM Razaz N John S . Maternal mortality in the United States: recent trends, current status, and future considerations. Obstet Gynecol. (2021) 137:763–71. doi: 10.1097/AOG.0000000000004361 33831914 PMC8055191

[17] The The Ministry of Health, P.R.China. National Work Manual for Maternal and Child Health Surveillance (2021 Edition). Available online at: http://www.mchscn.cn/National-22.html (Accessed December 24, 2021).

[18] World Health Organization. Maternal Death Surveillance and Response (MDSR): Operational Guidelines. Geneva: World Health Organization. (2017).

[19] World Health Organization. Maternal Deaths, Number. Geneva: World Health Organization. (2026).

[20] Liang J Li X Dai L Zeng W Li Q Li M . The changes in maternal mortality in 1000 counties in mid-Western China by a government-initiated intervention. PLoS ONE. (2012) 7:e37458. doi: 10.1371/journal.pone.0037458 22629398 PMC3357422

[21] Say L Chou D Gemmill A Tunçalp Ö Moller AB Daniels J . Global causes of maternal death: a WHO systematic analysis. Lancet Global Health. (2014) 2:e323–333. doi: 10.1016/S2214-109X(14)70227-X 25103301

[22] The The Ministry of Health P.R. China. Opinions on Promoting the Gradual Equalization of Basic Public Health Services. Available online at: https://www.gov.cn/gongbao/content/2010/content_1555969.htm (Accessed July 09, 2009).

[23] Zhao P Diao Y You L Wu S Yang L Liu Y. The influence of basic public health service project on maternal health services: an interrupted time series study. BMC Public Health. (2019) 19:824. doi: 10.1186/s12889-019-7207-1 31242879 PMC6595598

[24] The The Ministry of Health P.R.China. Circular on Strengthening the Work of Ensuring Maternal and Infant Safety. Available online at: https://www.gov.cn/gongbao/content/2018/content_5265003.htm (Accessed July 21, 2017).

[25] Rao Z Liu B Li D Qing Z Lu Y Yin D . Reducing urban-rural disparities in maternal and child mortality in China: a 33-year analysis and projection to 2030. J Glob Health. (2026) 16:04011. doi: 10.7189/jogh.16.04011 41524238 PMC12796867

[26] Wang W Zhang Y Lin B Mei Y Ping Z Zhang Z. The urban-rural disparity in the status. Int J Environ Res Public Health. (2020) 17:3848. doi: 10.3390/ijerph17113848 32485790 PMC7312746

[27] Alsabi FA Orabi AM Bajamal EZ. Knowledge and attitude of pregnant women about preeclampsia in King Abdulaziz medical City, Western region: a cross-sectional study. PLoS ONE. (2025) 20:e0312304. doi: 10.1371/journal.pone.0312304 40334247 PMC12058169

[28] Halmer S Fohleitner S Jutz F Klee S Busvine C Wichert-Schmitt B . Healthcare providers' awareness and management of cardiovascular risks in women with hypertensive disorders of pregnancy and gestational diabetes. Arch Gynecol Obstet. (2025) 312:413–23. doi: 10.1007/s00404-025-08012-8 40249408 PMC12334435

[29] Alipour J Payandeh A Karimi A. Prevalence of maternal mortality causes based on ICD-MM: a systematic review and meta-analysis. BMC Pregnancy Childbirth. (2023) 23:821. doi: 10.1186/s12884-023-06142-y 38017449 PMC10683107

[30] Li Y Zhang Y Fang S Liu S Liu X Li M . Analysis of inequality in maternal and child health outcomes and mortality from 2000 to 2013 in China. Int J Equity Health. (2017) 16:66. doi: 10.1186/s12939-017-0558-2 28427423 PMC5399313

[31] Chen L Feng P Shaver L Wang Z. Maternal mortality ratio in China from 1990 to 2019: trends, causes and correlations. BMC Public Health. (2021) 21:1536. doi: 10.1186/s12889-021-11557-3 34380436 PMC8359022

[32] Mesceriakova-Veliuliene O Kalediene R. Changes in mortality inequalities in urban and rural populations during 1990-2018: Lithuanian experience. Medicina (Kaunas, Lithuania) 2021, 57(8). doi: 10.3390/medicina57080750 PMC8398674 34440956

[33] Ma Y Xiao P Yu L Ni H Huang S Wang M . The allocation and fairness of health human resources in Chinese maternal and child health care institutions: a nationwide longitudinal study. BMC Health Serv Res. (2023) 23:151. doi: 10.1186/s12913-023-09076-5 36782193 PMC9926631

[34] Xu L You X Cui Y You J. Health poverty alleviation in China from the perspective of historical institutionalism: policy changes and driving factors. Front Public Health. (2023) 11:1265588. doi: 10.3389/fpubh.2023.1265588 38298260 PMC10829491

[35] Li D Yamada M Gao D Yang F Nie H. Spatial variations in health service utilization among migrant population: a perspective on health equity. Front Public Health. (2024) 12:1447723. doi: 10.3389/fpubh.2024.1447723 39440182 PMC11495394

[36] Gallos I Williams CR Price MJ Tobias A Devall A Allotey J . Prognostic accuracy of clinical markers of postpartum bleeding in predicting maternal mortality or severe morbidity: a WHO individual participant data meta-analysis. Lancet (London, England). (2025) 406:1969–82. doi: 10.2139/ssrn.5290994 41056961 PMC12549477

[37] Riches J Jafali J Twabi HH Chimwaza Y Onrust M Bilesi R . Avoidable factors associated with maternal death from postpartum haemorrhage: a national Malawian surveillance study. BMJ Global Health. (2025) 10:e015781. doi: 10.1136/bmjgh-2024-015781 PMC11748944 39788754

[38] Riches J Chimwaza Y Magreta Chakhame BI Milln J Twabi HH Bilesi R . BMJ Global Health (2024) 9:e016999. doi: 10.1136/bmjgh-2024-016999 PMC11590827 39581635

[39] Souza JP Day LT Rezende-Gomes AC Zhang J Mori R Baguiya A . A global analysis of the determinants of maternal health and transitions in maternal mortality. Lancet Global Health. (2024) 12:e306–16. doi: 10.1016/S2214-109X(23)00468-0 38070536

[40] Huang RS Spence AR Abenhaim HA. Non-obstetric maternal mortality trends by race in the United States. Matern Child Health J. (2024) 28:895–904. doi: 10.1007/s10995-023-03862-7 38147278

[41] Collier AY Molina RL. Maternal mortality in the United States: Updates on trends, causes, and solutions. Neoreviews. (2019) 20:e561–74. doi: 10.1542/neo.20-10-e561 31575778 PMC7377107

[42] Li HT Xue M Hellerstein S Cai Y Gao Y Zhang Y . Association of China's universal two child policy with changes in births and birth related health factors: national, descriptive comparative study. BMJ (Clin Res ed). (2019) 366:l4680. doi: 10.1136/bmj.l4680 PMC6699592 31434652

[43] Wang Y Kong F Fu Y Qiao J. How can China tackle its declining fertility rate? BMJ (Clin Res ed). (2024) 386:e078635. doi: 10.1136/bmj-2023-078635 PMC11359724 39214553

[44] Lean SC Derricott H Jones RL Heazell AEP. Advanced maternal age and adverse pregnancy outcomes: a systematic review and meta-analysis. PLoS ONE. (2017) 12:e0186287. doi: 10.1371/journal.pone.0186287 29040334 PMC5645107

[45] Bates SM Greer IA Middeldorp S Veenstra DL Prabulos AM Vandvik PO. VTE, thrombophilia, antithrombotic therapy, and pregnancy: antithrombotic therapy and prevention of thrombosis, 9th ed: American college of chest physicians evidence-based clinical practice guidelines. Chest. (2012) 141:e691S−736S. doi: 10.1378/chest.11-2300 PMC3278054 22315276

[46] Hernáez Á Skåra KH Page CM Mitter VR Hernández MH Magnus P . Parental genetically predicted liability for coronary heart disease and risk of adverse pregnancy outcomes: a cohort study. BMC Med. (2024) 22:35. doi: 10.1186/s12916-023-03223-9 38273336 PMC10809500

[47] Combellick JL Basile Ibrahim B Esmaeili A Phibbs CS Johnson AM Patton EW . Addressing the U.S. maternal health crisis: a systematic review of healthcare access barriers, disparate outcomes, and effective interventions. Int J Environ Res Public Health. (2023) 21:1814063. doi: 10.3389/fpubh.2026.1814063 PMC13149117 42110288

